# Evaluating infra-orbital nerve injury in zygomaticomaxillary complex fractures: a systematic review and meta-analysis

**DOI:** 10.3389/froh.2025.1726808

**Published:** 2025-12-05

**Authors:** Unnati Bimal Mehta, Uday Londhe, Kalyani Bhate, Manoj Bafna, Somya Pande, Lakshmi Shetty

**Affiliations:** Department of Oral and Maxillofacial Surgery, Dr DY Patil Vidyapeeth University Dr DY Patil Dental College and Hospital, Pune, India

**Keywords:** infraorbital nerve injury, paresthesia, sensory recovery, surgical management, zygomatico-maxillary complex fractures

## Abstract

**Aim:**

This systematic review aimed to assess infraorbital nerve (ION) injury in zygomatico-maxillary complex (ZMC) fractures, specifically examining the incidence, diagnostic approaches, and comparative efficacy of surgical vs. conservative management.

**Methods:**

This review was completed in accordance with PRISMA 2020 principles and was recorded in PROSPERO (CRD42024545221). The Cochrane CENTRAL, Embase, MEDLINE, and PubMed databases were thoroughly examined between January 2015 and April 2025. Adult patients (≥18 years) with radiologically confirmed ZMC fractures who specifically reported infraorbital nerve outcomes, such as sensory abnormalities, diagnostic evaluations, and recovery after therapy, were eligible for inclusion in the studies. Cochrane RoB 2.0 for randomized trials and the modified Newcastle-Ottawa Scale for observational studies were used to assess the quality of the studies. RevMan 5.4 was used to carry out statistical pooling using a random-effects model.

**Results:**

Initially, 457 articles were found in the literature search; duplicates were eliminated, titles and abstracts were screened, and 48 complete texts were examined; 13 studies (1,308 patients) met the inclusion criteria. The meta-analysis includes 11 trials with 802 patients. After ZMC fractures, the combined incidence of ION damage was 51.9% (95% CI: 48%–55%). When compared to conservative therapies, early surgical decompression (within two weeks after the damage) consistently produced superior sensory recovery. With little use of objective testing (blink reflex, current perception thresholds), diagnostic evaluations were mostly based on subjective measurements (two-point discrimination, pain thresholds).

**Conclusion:**

Infraorbital nerve injury remains a prevalent complication in ZMC fractures, affecting approximately half of the patients. Surgical decompression performed early after injury significantly improves sensory recovery outcomes over conservative management. There remains substantial variability in diagnostic methods and outcome measurements, highlighting the need for standardized approaches. Future randomized controlled trials with robust methodologies and consistent outcome assessments are necessary to enhance clinical guidance and patient outcomes.

**Systematic Review Registration:**

: ROSPERO CRD42024545221.

## Introduction

The zygomatico-maxillary complex (ZMC) plays a significant role in the contour and aesthetics of the face. Due to their prominence and the complexity of their anatomy, ZMC fractures are commonly involved in high-impact trauma, such as auto accidents, sports-related traumatic injury, assault, and falls (1). Among the various complications of ZMC fractures, damage to the infraorbital nerve (ION), a terminal branch of the maxillary branch of the trigeminal nerve, remains a serious concern (2). These injuries typically affect the lower eyelid, lateral aspect of the nose, upper lip, and cheek and manifest as paresthesia, hypoesthesia, anesthesia, or chronic neuropathic pain (3).

### Pathophysiology of infra-orbital nerve injury

Infraorbital nerve (ION) damage following zygomatico-maxillary complex (ZMC) fractures can occur through several mechanisms—direct nerve transection, compression by displaced bone fragments, or swelling due to the body's inflammatory response (4). Despite advancements in imaging and surgical techniques, reported rates of sensory disturbances after such fractures remain highly variable, ranging from 20% to as high as 80% across different studies. High-resolution CT scans are invaluable for spotting fracture patterns, nerve entrapment, or bony compression. Clinically, however, assessment largely hinges on subjective methods such as light touch, pinprick, and two-point discrimination tests (5–7).

Management of ION injury is highly individualized, depending on the extent of nerve involvement and patient-specific factors. Options range from watchful waiting to timely surgical decompression. Persistent sensory loss doesn't just affect physical function—it can take a toll on a patient's psychological well-being, underlining the importance of early recognition and tailored intervention (8, 9).

Yet, despite a growing body of research on this topic, findings remain inconsistent. Variability in patient populations, diagnostic criteria, and outcome measures has led to considerable heterogeneity across studies. Because of this, there's still no clear consensus on the true prevalence of ION injury, the most accurate diagnostic tools, or the most effective treatment strategies. These challenges highlight the need for a well-conducted systematic review to synthesize the current evidence, evaluate diagnostic and therapeutic approaches, and provide clearer, evidence-based guidance for managing patients with ZMC-related ION injuries. Hence, in this study, we aim to review the current evidence on the frequency, diagnosis, treatment of infraorbital nerve injuries associated with zygomatico-maxillary complex fractures.

### Objectives

1. To determine the incidence and prevalence of ION injuries in patients with ZMC fractures.
2. To evaluate the diagnostic accuracy of clinical and radiological methods for detecting ION injury.
3. To compare the effectiveness of conservative vs. surgical management strategies in restoring nerve function.

## Materials and methods

### Study design

This systematic review and meta-analysis was conducted in accordance with the Preferred Reporting Items for Systematic Reviews and Meta-Analyses (PRISMA) 2020 guidelines (10). The protocol was registered with the International Prospective Register of Systematic Reviews (PROSPERO) under registration number **CRD42024545221** (11). Since the study was based solely on previously published data, ethical approval was not required.

### Data sources and search strategy

A comprehensive literature search was conducted using the following databases: PubMed, MEDLINE, EMBASE, Cochrane Central Register of Controlled Trials (CENTRAL).

The final search was executed on **May 1, 2025**, and included studies published from **January 2015 to April 2025**. All keyword categories were combined using the Boolean operator “**AND**” to narrow the search results (for example, “*zygomatico-maxillary complex fracture*” *AND “infra-orbital nerve injury” AND “sensory recovery”*). Within each concept group, related terms and synonyms were connected using “**OR**”.

The search was limited to the title and abstract fields in each database so that only studies directly focused on the topic were retrieved, avoiding those that mentioned the terms only in passing.

Additionally, the reference lists of all included studies were manually screened to identify any additional relevant publications.

### Eligibility criteria

#### Inclusion criteria

1. Original studies (randomized controlled trials, prospective or retrospective cohorts, and cross-sectional studies).
2. Studies involving adult patients (≥18 years) with ZMC fractures confirmed by clinical and radiological assessment.
3. Studies reporting at least one outcome related to infraorbital nerve (ION) function, such as sensory disturbances, recovery, or treatment outcomes.
4. Only studies reporting follow-up of at least 3 months or clearly defined timelines for sensory recovery were included to permit temporal comparison of ION healing patterns

#### Exclusion criteria

1. Case reports, letters to the editor, technical notes, or editorials without original patient data.
2. Studies unrelated to infraorbital nerve involvement or not specific to ZMC fractures.
3. Non-English studies without accessible English translations.
4. Animal, cadaveric, or laboratory-based studies.
5. Studies published prior to January 2015.

#### Study selection and data extraction

Two reviewers independently screened all titles and abstracts, followed by full-text assessments for eligibility. Discrepancies were resolved through discussion with a third reviewer. Data were extracted using a standardized data extraction form and included (Table 1):

• Study characteristics: Author, publication year, country, study design
• Patient demographics: Sample size, age, sex
• Fracture characteristics: Classification, mechanism of injury
• Diagnostic methods: Clinical testing, radiological imaging
• Treatment interventions: Surgical (e.g., ORIF, decompression) vs. conservative management
• Outcome measures: Prevalence and type of ION injury, sensory recovery, time to recovery, nerve conduction findings, quality of life
• Secondary outcomes: Complications, psychosocial impact, and predictors of recovery

**Table 1:** PICO framework (14).

Element	Description
Population	Adults (≥18 years) with ZMC fractures confirmed on imaging, exhibiting infraorbital nerve injury.
Intervention	Surgical management, including ORIF, decompression, or nerve exploration.
Comparison	Conservative management, including observation, analgesics, or corticosteroids.
Outcomes	Primary: Incidence of ION injury, sensory recovery, time to recovery, nerve conduction changes, functional recovery (e.g., VAS, Facial Disability Index). Secondary: Psychosocial effects, complications, reoperation rates, and predictors of recovery (e.g., age, fracture type, timing).

### Risk of bias assessment

The Joanna Briggs Institute (JBI) (12) critical appraisal tool was used to evaluate the quality of observational and cross-sectional studies, assessing domains such as participant selection, exposure measurement, confounding, and outcome reporting. For randomized controlled trials, the Cochrane Risk of Bias 2.0 tool was applied, examining key domains including randomization, deviations from intended interventions, missing outcome data, measurement of outcomes, and selective reporting (13). Studies were categorized as having low, moderate, or high risk of bias based on these assessments. All evaluations were performed independently by two reviewers, with consensus on discrepancies.

The inclusion of studies was guided by transparent and predefined quality parameters. Only studies with appropriate participant recruitment, clearly defined exposure and outcome measures, adequate control for potential confounders, and sufficient follow-up were included. Studies were excluded if they lacked detailed patient-level data on infraorbital nerve outcomes, employed non-standardized or poorly validated diagnostic tools, or provided incomplete follow-up information. All evaluations were independently conducted by two reviewers, and any disagreements were resolved by consensus.

### Statistical analysis

A single-arm meta-analysis was performed using Review Manager (RevMan) version 5.4 to pool the proportions of infraorbital nerve dysfunction across studies. Since the outcome data were sufficiently homogeneous, the pooled proportion was calculated, and a random-effects model was applied to account for clinical and methodological heterogeneity. Due to the lack of control group data, we did not calculate risk ratios (RR) or odds ratios. Heterogeneity was not assessed using the *I*^2^ statistic or Cochrane's Q test, as they are not applicable for single-arm data.

## Results

**Figure 1:**
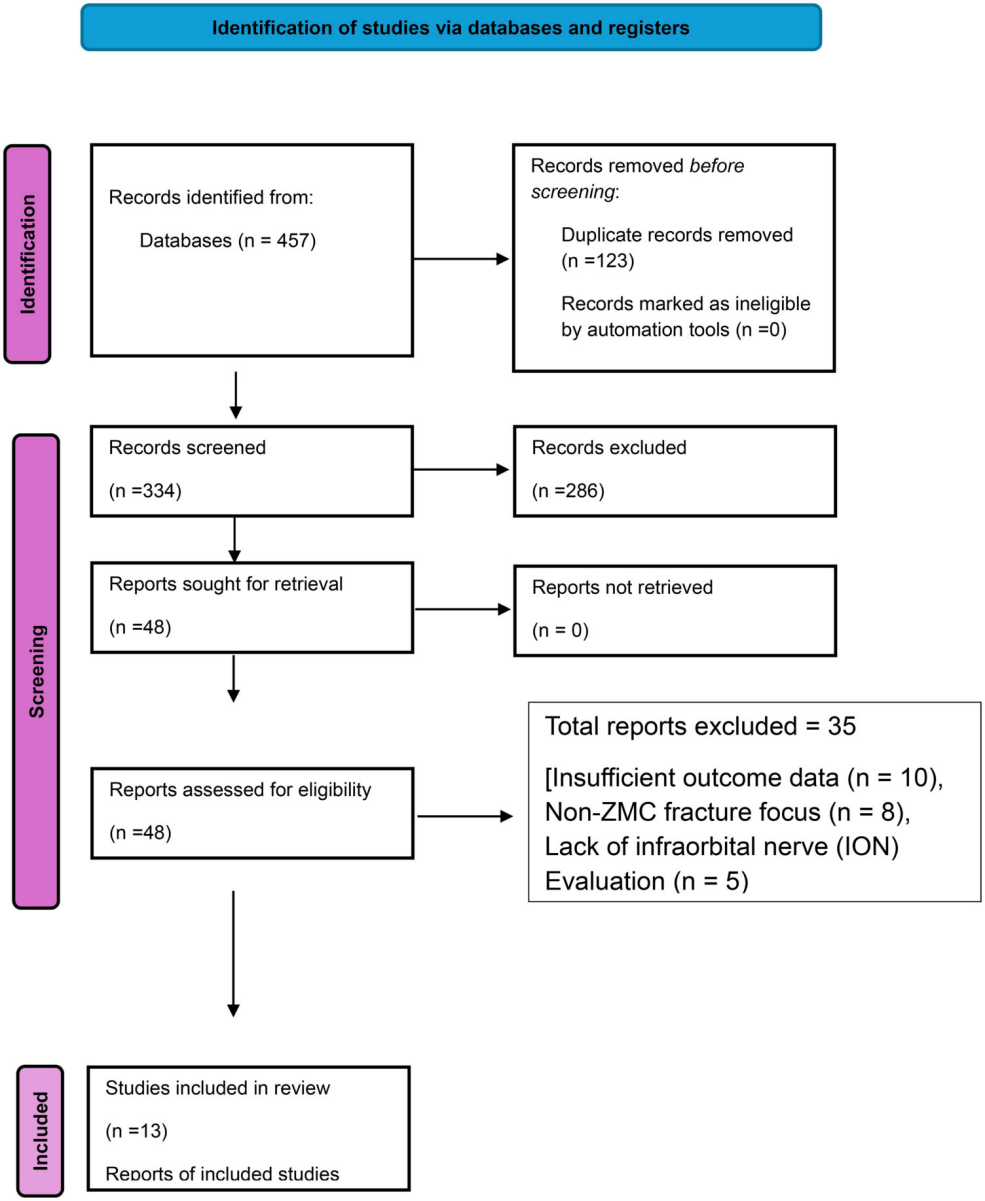
PRISMA flow diagram.

The initial database search identified 457 records, of which 123 were duplicates and removed. After screening the titles and abstracts of the remaining 334 articles, 286 were excluded for not meeting the inclusion criteria. A total of 48 full-text articles were then assessed for eligibility, resulting in the exclusion of 35 studies due to insufficient outcome data (*n* = 10), non-ZMC fracture focus (*n* = 8), lack of infraorbital nerve (ION) evaluation (*n* = 5), or other reasons (*n* = 12). Thirteen studies met the eligibility criteria and were included in this systematic review. Of these, 11 studies were suitable for quantitative synthesis (meta-analysis). The study selection process is illustrated in Figure 1.

Thirteen studies comprising 1,308 patients with zygomatico-maxillary complex fractures were included (Table 2). Reported incidence of infraorbital nerve dysfunction ranged from 25% to 79%, with higher rates noted in displaced fractures and conservatively managed cases [Dubron et al. (4), Arun et al. (15)]. Most studies relied on clinical assessment methods like two-point discrimination and patient-reported symptoms, while a few incorporated objective tests such as blink reflex and current perception threshold [Okochi et al. (16), Park et al. (17)]. CT imaging was routinely used to evaluate fracture displacement. Management strategies varied; minimally displaced fractures were often managed conservatively, while surgical intervention (ORIF ± decompression) was preferred for severe cases. Surgical management generally led to better sensory recovery [Baloch et al. (18)], although persistent deficits were reported even post-surgery [Taylor et al. (19)].

**Table 2:** Final summary table of included studies (*n* = 13).

Author (Year)	Country	Study design	Sample size	Treatment type	ION outcome measure	Follow-up duration	Included in SR	Included in MA
Arun et al. (15)	India	Prospective Observational	103	Non-surgical	Paresthesia (6 parameters)	6 months	Yes	Yes
Baloch et al. (18)	Pakistan	Randomized Controlled Trial	128	Open vs. Closed Reduction	Neurosensory recovery	24 weeks	Yes	Yes
Bashiri et al. (20)	Iran	Controlled Clinical Trial	71	Photobiomodulation	VAS, TPD, Pain scale	3 months	Yes	No
Das et al. (21)	India	Comparative Clinical Study	13	ORIF, Reduction, Conservative	Mechanical, heat, pain threshold	6 months	Yes	Yes
Dhabaria et al. (22)	India	Prospective Evaluative Study	13	ORIF (2-point fixation)	TPD, Pain, QoL	6 months	Yes	Yes
Dubron et al. (4)	Belgium	Retrospective Cohort	272	Mixed	ION hypoaesthesia, predictors	4.4 months avg.	Yes	Yes
Homer et al. (23)	USA	Prospective Pilot Study	42 (ZMC subset)	Surgical vs. Conservative (ZMC)	Grading scale for sensory dysfunction	21.7 months Avg.	Yes	Yes (ZMC group only)
Ishaq et al. (24)	Pakistan	Randomized Controlled Trial	100 (50 per group)	Open vs. Closed Reduction	Sharp prick test, symptom relief	3 months	Yes	Yes
Okochi et al. (16)	Japan	Retrospective Cohort	10	ORIF with decompression	S-W test, CPT (A*β*, A*δ*, C)	5 years	Yes	Yes
Park et al. (17)	South Korea	Prospective Observational	18	ORIF ± Orbital floor recon	TPD, VAS, blink reflex	4.7 months avg	Yes	Yes
Tabrizi et al. (25)	Iran	Cross-sectional	40	ORIF	TPD, Brush test, VAS	6 months	Yes	Yes
Taylor et al. (19)	UK	Retrospective Review	432 (20 reop.)	ORIF/Closed Reduction	ION discomfort/anecdotal mention; not formally assessed	≥3 months (avg. 4.7 yrs)	Yes	No
Yoon et al. (26)	South Korea	Retrospective Cohort	166	ORIF ± Decompression	VAS	6 months	Yes	Yes

### Meta-analysis

Out of the 13 studies included in the systematic review, 11 studies (*n* = 802 patients) were included in the meta-analysis. Two studies were excluded: Taylor et al. (19) for lacking formal infraorbital nerve dysfunction assessment, and Bashiri et al. (20) for focusing on treatment efficacy rather than baseline incidence.

A single-arm meta-analysis was conducted pooling proportions of patients experiencing infraorbital nerve dysfunction following ZMC fractures. The pooled proportion of infraorbital nerve dysfunction across studies was 51.9% (95% CI: 48%–55%) (Table 3). Due to the absence of consistently reported control group data across the included studies, computation of comparative risk differences, odds ratios, or relative risks was not feasible. As a result, forest plots and funnel plots generated in RevMan 5.4 demonstrated “Not estimable” outcomes for risk ratios. However, incidence-based pooling was achieved based on proportion analysis.

**Table 3:** Incidence proportions of infraorbital nerve dysfunction in ZMC fractures: a summary of studies.

Study	Events	Total	Proportion (%)	95% CI (Approximate)
Yoon et al. (26)	120	166	72.3%	(65%–78%)
Tabrizi et al. (25)	31	40	77.5%	(62%–88%)
Park et al. (17)	2	18	11.1%	(1%–35%)
Okochi et al. (16)	10	10	100%	(70%–100%)
Ishaq et al. (24)	37	100	37.0%	(27%–48%)
Homer et al. (23)	20	42	47.6%	(32%–63%)
Dubron et al. (4)	100	272	36.8%	(31%–42%)
Dhabaria et al. (22)	2	13	15.4%	(2%–45%)
Das et al. (21)	3	13	23.1%	(5%–54%)
Baloch et al. (18)	65	128	50.8%	(42%–59%)
Arun et al. (15)	27	103	26.2%	(18%–36%)

**Figure 2:**
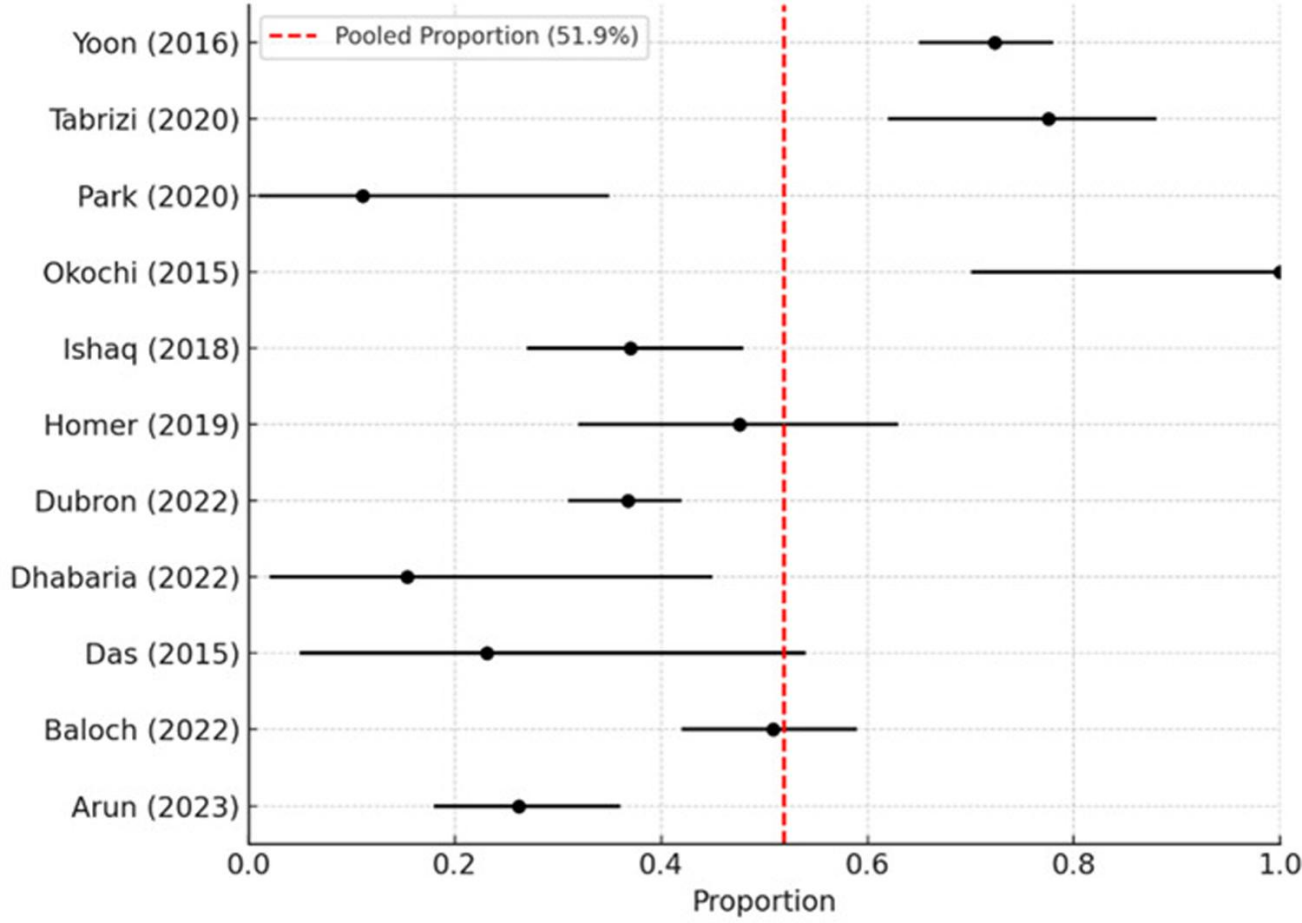
Forest plot showing the pooled incidence proportion of infraorbital nerve dysfunction in patients with zygomatico-maxillary complex fractures (overall = 51.9%).

Figure 2 illustrates the forest plot summarizing the pooled proportion of infraorbital nerve dysfunction following zygomatico-maxillary complex fractures across the included studies. Each horizontal line represents the 95% confidence interval for an individual study, and the red dashed line indicates the overall pooled estimate (51.9%).

### Risk of bias

The two randomized controlled trials [Baloch et al. (18), Ishaq et al. (24)] were assessed using the RoB 2 tool. Baloch et al. (18) had moderate overall risk due to concerns in randomization and outcome measurement, while Ishaq et al. (24) had a high risk mainly due to poor randomization processes (Table 4). The observational and cohort studies were assessed using modified Newcastle-Ottawa Scale criteria (27); most showed low risk across domains, with a few [Dubron et al. (4), Taylor et al. (19), Das et al. (21)] rated as moderate risk due to sample representativeness, confounding, or incomplete reporting (Table 5).

**Table 4:** Risk of bias assessment for randomized controlled trials (RCTs).

Study	D1: randomization process	D2: deviations from intended interventions	D3: missing outcome data	D4: measurement of outcome	D5: selection of reported results	Overall risk of bias
Baloch et al. (18)	Some concerns	Low risk	Low risk	Some concerns	Low risk	Moderate
Ishaq et al. (24)	High risk	Some concerns	Low risk	Some concerns	Low risk	High

**Table 5:** Risk of bias assessment for observational and cohort studies.

Study	Sample representativeness	Confounding factors addressed	Outcome measurement method	Follow-up adequate?	Reporting transparency	Overall risk of bias
Arun et al. (15)	Low risk	Low risk	Low risk	Yes	Clear	Low
Bashiri et al. (20)	Low risk	Low risk	Low risk	Yes	Clear	Low
Das et al. (21)	Some concerns	Moderate risk	Low risk	Yes	Clear	Moderate
Dhabaria et al. (22)	Low risk	Low risk	Low risk	Yes	Clear	Low
Dubron et al. (4)	Some concerns	Some concerns	Low risk	Yes	Clear	Moderate
Homer et al. (23)	Low risk	Low risk	Low risk	Yes	Clear	Low
Okochi et al. (16)	Low risk	Low risk	Low risk	Yes	Clear	Low
Park et al. (17)	Low risk	Low risk	Low risk	Yes	Clear	Low
Tabrizi et al. (25)	Low risk	Low risk	Low risk	Yes	Clear	Low
Taylor et al. (19)	Some concerns	Moderate risk	Low risk	Yes	Incomplete outcome detail	Moderate
Yoon et al. (26)	Low risk	Low risk	Low risk	Yes	Clear	Low

## Discussion

This meta-analysis and systematic review offer a current assessment of the prevalence, diagnosis, and treatment approaches for infraorbital nerve (ION) dysfunction after zygomatico-maxillary complex (ZMC) fractures. Sensory abnormalities are a significant clinical problem following midfacial trauma, as seen by the pooled prevalence of infraorbital nerve impairment (∼51.9%).

The results of Dubron et al. (4), who documented a 37.3% incidence of infraorbital nerve hypoaesthesia after ZMC fractures, especially when the fracture line crossed the infraorbital canal, are in keeping with our findings (4). According to Dhabaria et al. (22), neurosensory problems are also common; over 80% of patients initially exhibit deficiencies but significantly recover after open reduction and internal fixation (ORIF) (22).

In our quantitative synthesis, studies such as Ishaq et al. (24) and Baloch et al. (18) demonstrated markedly improved sensory recovery among patients who underwent decompression within the first two weeks, compared to those managed conservatively or with delayed surgery. These findings substantiate that early intervention during the acute phase prevents prolonged nerve compression and enhances neurosensory restitution. The results of our review were less positive, confirming the findings of Arun et al. (15), who observed persisting infraorbital nerve impairments in patients treated conservatively (15). Conservative care was mainly saved for mildly displaced fractures.

Accurate diagnosis is still difficult to achieve. Only a small number of the included studies used objective clinical measures like blink reflex and current perception threshold (CPT), but subjective tests like two-point discrimination and pain thresholds were more frequently used [Okochi et al. (16), Park et al. (17)]. Like Dubron et al. (4), high-resolution CT imaging was frequently used for fracture assessment, although its predictive power for sensory outcomes was not always reliable (4).

Even with proper surgical treatment, persistent sensory abnormalities can have serious psychosocial repercussions that affect speech, facial expression, and overall quality of life. Compared to isolated orbital fractures, ZMC fractures typically have worse long-term sensory results, and recovery could not be full even with intervention, as noted by Homer et al. (23).

Although the present meta-analysis focused on studies with detailed neurosensory evaluation, it is noteworthy that recent epidemiological data by Pande et al. (28) also highlighted the persistence of infraorbital nerve symptoms in ZMC fractures. While their retrospective cohort did not provide quantitative nerve recovery timelines or standardized sensory assessments—hence, it was excluded from the pooled analysis—the authors observed that a subset of patients continued to report paraesthesia even after surgical fixation. This observation, although limited, reinforces our findings that anatomical reduction alone does not guarantee neurosensory restoration. Rather, the timing of intervention and degree of nerve decompression are critical determinants of sensory recovery, further emphasizing the need for structured postoperative neurosensory follow-up in future studies.

Although they have not yet been thoroughly studied in large-scale trials, emerging technologies such as intraoperative nerve monitoring and three-dimensional imaging have the potential to improve evaluation and management. To more accurately evaluate functional and psychological recovery, future research should prioritize standardized outcome measures and include patient-reported outcome measures (PROMs).

Future studies should aim to use consistent and objective methods for sensory evaluation, combining quantitative sensory testing techniques such as current perception threshold and blink reflex with well-validated clinical tools like two-point discrimination and Semmes–Weinstein monofilament testing. Maintaining uniform follow-up intervals and incorporating patient-reported outcome measures will help generate more comparable data across studies and strengthen the quality of future meta-analysis.

### Limitations

This systematic review has a number of drawbacks. Variations in fracture categorization systems, infraorbital nerve dysfunction diagnostic methods, and follow-up intervals across the included studies contributed moderate variability. Internal validity may have been impacted by the observational design of a significant percentage of research, which has intrinsic concerns of selection bias, ambiguous blinding, and insufficient outcome reporting. Furthermore, a number of studies' small sample sizes might have underpowered some subgroup analyses, reducing the conclusions’ ability to be applied broadly. The exclusion of pertinent data published in other languages may have resulted from the linguistic restriction to English-only studies. Additionally, consistent data synthesis and comparison were made more difficult by the variations in neurosensory outcome evaluation techniques used in different investigations.

## Conclusion

Infraorbital nerve dysfunction remains a significant outcome of ZMC fractures, with a pooled incidence of 51.9%. Early surgical intervention, particularly within the first two weeks, has shown superior sensory recovery compared to delayed or conservative care. In clinical decision-making, ORIF is reserved for fractures with two- or three-point displacement, restricted ocular movements, enophthalmos/exophthalmos, or evident facial deformity. In contrast, closed reduction without plating is considered in undisplaced fractures or cases presenting primarily with nerve paraesthesia or entrapment without structural displacement. These distinctions reinforce that treatment must be guided by functional impairment and anatomical disruption rather than fracture presence alone.

## Data Availability

The original contributions presented in the study are included in the article/Supplementary Material, further inquiries can be directed to the corresponding author/s.
